# Supplementary material for: COZOID: contact zone identifier for visual analysis of protein-protein interactions
Source: BMC Bioinformatics. 2018 Apr 6;19:125. doi: 10.1186/s12859-018-2113-6 (PMC5889581; doi:10.1186/s12859-018-2113-6)
Supplement: Supplementary file 2 — Software build. Executable binary file of the software tool. (ZIP 118,784 kb) [file 12859_2018_2113_MOESM2_ESM.zip › cozoid/platform/docs/testng-6.5.1-javadoc.zip!/org/testng/TestRunner.html]

TestRunner (TestNG)


---


|  |  |  |
| --- | --- | --- |
| **Overview** \| **Package** \| **Class** \| **Tree** \| **Deprecated** \| **Index** \| **Help** | |  |
| **PREV CLASS**   **NEXT CLASS** | **FRAMES**    **NO FRAMES**     **All Classes** |  |
| SUMMARY: NESTED \| FIELD \| CONSTR \| METHOD | DETAIL: FIELD \| CONSTR \| METHOD |  |


---


## org.testng Class TestRunner

```
java.lang.Object
  org.testng.TestRunner
```

**All Implemented Interfaces:**: java.io.Serializable, IAttributes, org.testng.internal.ITestResultNotifier, org.testng.internal.thread.graph.IThreadWorkerFactory<ITestNGMethod>, ITestContext

---

``` public class TestRunner extends java.lang.Object implements ITestContext, org.testng.internal.ITestResultNotifier, org.testng.internal.thread.graph.IThreadWorkerFactory<ITestNGMethod> ```

This class takes care of running one Test.

**Author:**
:   Cedric Beust, Apr 26, 2004

**See Also:**: Serialized Form

---

| **Constructor Summary** | |
| --- | --- |
|  | `TestRunner(org.testng.internal.IConfiguration configuration, ISuite suite, XmlTest test, boolean skipFailedInvocationCounts, java.util.List<IInvokedMethodListener> listeners)` |
| `protected` | `TestRunner(org.testng.internal.IConfiguration configuration, ISuite suite, XmlTest test, java.lang.String outputDirectory, org.testng.internal.annotations.IAnnotationFinder finder, boolean skipFailedInvocationCounts, java.util.List<IInvokedMethodListener> invokedMethodListeners)` |


| **Method Summary** | |
| --- | --- |
| `void` | `addFailedButWithinSuccessPercentageTest(ITestNGMethod testMethod, ITestResult result)` |
| `void` | `addFailedTest(ITestNGMethod testMethod, ITestResult result)` |
| `void` | `addGuiceModule(java.lang.Class<? extends com.google.inject.Module> cls, com.google.inject.Module module)` |
| `void` | `addInjector(java.util.List<com.google.inject.Module> moduleInstances, com.google.inject.Injector injector)` |
| `void` | `addInvokedMethod(org.testng.internal.InvokedMethod im)` |
| `void` | `addListener(java.lang.Object listener)` |
| `void` | `addPassedTest(ITestNGMethod tm, ITestResult tr)` |
| `void` | `addSkippedTest(ITestNGMethod tm, ITestResult tr)` |
| `void` | `addTestListener(ITestListener il)` |
| `java.util.List<org.testng.internal.thread.graph.IWorker<ITestNGMethod>>` | `createWorkers(java.util.List<ITestNGMethod> methods)`             Create a list of workers to run the methods passed in parameter. |
| `ITestNGMethod[]` | `getAfterSuiteMethods()` |
| `ITestNGMethod[]` | `getAfterTestConfigurationMethods()` |
| `ITestNGMethod[]` | `getAllTestMethods()` |
| `java.lang.Object` | `getAttribute(java.lang.String name)` |
| `java.util.Set<java.lang.String>` | `getAttributeNames()` |
| `ITestNGMethod[]` | `getBeforeSuiteMethods()` |
| `ITestNGMethod[]` | `getBeforeTestConfigurationMethods()` |
| `java.util.List<IConfigurationListener>` | `getConfigurationListeners()` |
| `XmlTest` | `getCurrentXmlTest()` |
| `java.util.Date` | `getEndDate()`             When this test stopped running. |
| `java.lang.String[]` | `getExcludedGroups()` |
| `java.util.Collection<ITestNGMethod>` | `getExcludedMethods()` |
| `IResultMap` | `getFailedButWithinSuccessPercentageTests()` |
| `IResultMap` | `getFailedConfigurations()`             Retrieves information about the failed configuration method invocations. |
| `IResultMap` | `getFailedTests()` |
| `java.util.Set<ITestResult>` | `getFailedTests(ITestNGMethod tm)` |
| `java.util.List<com.google.inject.Module>` | `getGuiceModules(java.lang.Class<? extends com.google.inject.Module> cls)` |
| `java.lang.String` | `getHost()` |
| `java.lang.String[]` | `getIncludedGroups()` |
| `com.google.inject.Injector` | `getInjector(java.util.List<com.google.inject.Module> moduleInstances)` |
| `java.util.List<ITestNGMethod>` | `getInvokedMethods()` |
| `org.testng.internal.IInvoker` | `getInvoker()` |
| `java.lang.String` | `getName()`             The name of this test. |
| `java.lang.String` | `getOutputDirectory()` |
| `IResultMap` | `getPassedConfigurations()`             Retrieves information about the successful configuration method invocations. |
| `IResultMap` | `getPassedTests()` |
| `java.util.Set<ITestResult>` | `getPassedTests(ITestNGMethod tm)` |
| `IResultMap` | `getSkippedConfigurations()`             Retrieves information about the skipped configuration method invocations. |
| `IResultMap` | `getSkippedTests()` |
| `java.util.Set<ITestResult>` | `getSkippedTests(ITestNGMethod tm)` |
| `java.util.Date` | `getStartDate()`             When this test started running. |
| `ISuite` | `getSuite()` |
| `XmlTest` | `getTest()` |
| `java.util.Collection<ITestClass>` | `getTestClasses()` |
| `java.util.List<ITestListener>` | `getTestListeners()` |
| `static int` | `getVerbose()` |
| `java.lang.Object` | `removeAttribute(java.lang.String name)`             Remove the attribute |
| `void` | `run()`             The main entry method for TestRunner. |
| `void` | `setAttribute(java.lang.String name, java.lang.Object value)`             Set a custom attribute. |
| `void` | `setMethodInterceptor(IMethodInterceptor methodInterceptor)` |
| `void` | `setOutputDirectory(java.lang.String od)` |
| `void` | `setTestName(java.lang.String name)` |
| `void` | `setVerbose(int n)` |

**Methods inherited from class java.lang.Object**

`clone, equals, finalize, getClass, hashCode, notify, notifyAll, toString, wait, wait, wait`

**Constructor Detail**

### TestRunner

```
protected TestRunner(org.testng.internal.IConfiguration configuration,
                     ISuite suite,
                     XmlTest test,
                     java.lang.String outputDirectory,
                     org.testng.internal.annotations.IAnnotationFinder finder,
                     boolean skipFailedInvocationCounts,
                     java.util.List<IInvokedMethodListener> invokedMethodListeners)
```

---


### TestRunner

```
public TestRunner(org.testng.internal.IConfiguration configuration,
                  ISuite suite,
                  XmlTest test,
                  boolean skipFailedInvocationCounts,
                  java.util.List<IInvokedMethodListener> listeners)
```


**Method Detail**

### getInvoker

```
public org.testng.internal.IInvoker getInvoker()
```

---


### getBeforeSuiteMethods

```
public ITestNGMethod[] getBeforeSuiteMethods()
```

---


### getAfterSuiteMethods

```
public ITestNGMethod[] getAfterSuiteMethods()
```

---


### getBeforeTestConfigurationMethods

```
public ITestNGMethod[] getBeforeTestConfigurationMethods()
```

---


### getAfterTestConfigurationMethods

```
public ITestNGMethod[] getAfterTestConfigurationMethods()
```

---


### getTestClasses

```
public java.util.Collection<ITestClass> getTestClasses()
```

---


### setTestName

```
public void setTestName(java.lang.String name)
```

---


### setOutputDirectory

```
public void setOutputDirectory(java.lang.String od)
```

---


### run

```
public void run()
```

:   The main entry method for TestRunner.
    This is where all the hard work is done:
    - Invoke configuration methods
    - Invoke test methods
    - Catch exceptions
    - Collect results
    - Invoke listeners
    - etc...

---


### createWorkers

```
public java.util.List<org.testng.internal.thread.graph.IWorker<ITestNGMethod>> createWorkers(java.util.List<ITestNGMethod> methods)
```

:   Create a list of workers to run the methods passed in parameter.
    Each test method is run in its own worker except in the following cases:
    - The method belongs to a class that has @Test(sequential=true)
    - The parallel attribute is set to "classes"
    In both these cases, all the methods belonging to that class will then
    be put in the same worker in order to run in the same thread.

    :   **Specified by:**: `createWorkers` in interface `org.testng.internal.thread.graph.IThreadWorkerFactory<ITestNGMethod>`

---


### getName

```
public java.lang.String getName()
```

:   **Description copied from interface: `ITestContext`**
:   The name of this test.

    :   **Specified by:**: `getName` in interface `ITestContext`

---


### getStartDate

```
public java.util.Date getStartDate()
```

:   **Description copied from interface: `ITestContext`**
:   When this test started running.

    :   **Specified by:**: `getStartDate` in interface `ITestContext`
    :   **Returns:**: Returns the startDate.

---


### getEndDate

```
public java.util.Date getEndDate()
```

:   **Description copied from interface: `ITestContext`**
:   When this test stopped running.

    :   **Specified by:**: `getEndDate` in interface `ITestContext`
    :   **Returns:**: Returns the endDate.

---


### getPassedTests

```
public IResultMap getPassedTests()
```

:   **Specified by:**: `getPassedTests` in interface `ITestContext`
:   **Returns:**: A list of all the tests that run successfully.

---


### getSkippedTests

```
public IResultMap getSkippedTests()
```

:   **Specified by:**: `getSkippedTests` in interface `ITestContext`
:   **Returns:**: A list of all the tests that were skipped

---


### getFailedTests

```
public IResultMap getFailedTests()
```

:   **Specified by:**: `getFailedTests` in interface `ITestContext`
:   **Returns:**: A map of all the tests that passed, indexed by their ITextMethor. **See Also:**: `ITestNGMethod`

---


### getFailedButWithinSuccessPercentageTests

```
public IResultMap getFailedButWithinSuccessPercentageTests()
```

:   **Specified by:**: `getFailedButWithinSuccessPercentageTests` in interface `ITestContext`
:   **Returns:**: A list of all the tests that failed but are being ignored because annotated with a successPercentage.

---


### getIncludedGroups

```
public java.lang.String[] getIncludedGroups()
```

:   **Specified by:**: `getIncludedGroups` in interface `ITestContext`
:   **Returns:**: All the groups that are included for this test run.

---


### getExcludedGroups

```
public java.lang.String[] getExcludedGroups()
```

:   **Specified by:**: `getExcludedGroups` in interface `ITestContext`
:   **Returns:**: All the groups that are excluded for this test run.

---


### getOutputDirectory

```
public java.lang.String getOutputDirectory()
```

:   **Specified by:**: `getOutputDirectory` in interface `ITestContext`
:   **Returns:**: Where the reports will be generated.

---


### getSuite

```
public ISuite getSuite()
```

:   **Specified by:**: `getSuite` in interface `ITestContext`
:   **Returns:**: Returns the suite.

---


### getAllTestMethods

```
public ITestNGMethod[] getAllTestMethods()
```

:   **Specified by:**: `getAllTestMethods` in interface `ITestContext`
:   **Returns:**: All the test methods that were run.

---


### getHost

```
public java.lang.String getHost()
```

:   **Specified by:**: `getHost` in interface `ITestContext`
:   **Returns:**: The host where this test was run, or null if it was run locally. The returned string has the form: host:port

---


### getExcludedMethods

```
public java.util.Collection<ITestNGMethod> getExcludedMethods()
```

:   **Specified by:**: `getExcludedMethods` in interface `ITestContext`
:   **Returns:**: All the methods that were not included in this test run.

---


### getFailedConfigurations

```
public IResultMap getFailedConfigurations()
```

:   **Description copied from interface: `ITestContext`**
:   Retrieves information about the failed configuration method invocations.

    :   **Specified by:**: `getFailedConfigurations` in interface `ITestContext`
    :   **See Also:**: `ITestContext.getFailedConfigurations()`

---


### getPassedConfigurations

```
public IResultMap getPassedConfigurations()
```

:   **Description copied from interface: `ITestContext`**
:   Retrieves information about the successful configuration method invocations.

    :   **Specified by:**: `getPassedConfigurations` in interface `ITestContext`
    :   **See Also:**: `ITestContext.getPassedConfigurations()`

---


### getSkippedConfigurations

```
public IResultMap getSkippedConfigurations()
```

:   **Description copied from interface: `ITestContext`**
:   Retrieves information about the skipped configuration method invocations.

    :   **Specified by:**: `getSkippedConfigurations` in interface `ITestContext`
    :   **See Also:**: `ITestContext.getSkippedConfigurations()`

---


### addPassedTest

```
public void addPassedTest(ITestNGMethod tm,
                          ITestResult tr)
```

:   **Specified by:**: `addPassedTest` in interface `org.testng.internal.ITestResultNotifier`

---


### getPassedTests

```
public java.util.Set<ITestResult> getPassedTests(ITestNGMethod tm)
```

:   **Specified by:**: `getPassedTests` in interface `org.testng.internal.ITestResultNotifier`

---


### getFailedTests

```
public java.util.Set<ITestResult> getFailedTests(ITestNGMethod tm)
```

:   **Specified by:**: `getFailedTests` in interface `org.testng.internal.ITestResultNotifier`

---


### getSkippedTests

```
public java.util.Set<ITestResult> getSkippedTests(ITestNGMethod tm)
```

:   **Specified by:**: `getSkippedTests` in interface `org.testng.internal.ITestResultNotifier`

---


### addSkippedTest

```
public void addSkippedTest(ITestNGMethod tm,
                           ITestResult tr)
```

:   **Specified by:**: `addSkippedTest` in interface `org.testng.internal.ITestResultNotifier`

---


### addInvokedMethod

```
public void addInvokedMethod(org.testng.internal.InvokedMethod im)
```

:   **Specified by:**: `addInvokedMethod` in interface `org.testng.internal.ITestResultNotifier`

---


### addFailedTest

```
public void addFailedTest(ITestNGMethod testMethod,
                          ITestResult result)
```

:   **Specified by:**: `addFailedTest` in interface `org.testng.internal.ITestResultNotifier`

---


### addFailedButWithinSuccessPercentageTest

```
public void addFailedButWithinSuccessPercentageTest(ITestNGMethod testMethod,
                                                    ITestResult result)
```

:   **Specified by:**: `addFailedButWithinSuccessPercentageTest` in interface `org.testng.internal.ITestResultNotifier`

---


### getTest

```
public XmlTest getTest()
```

:   **Specified by:**: `getTest` in interface `org.testng.internal.ITestResultNotifier`

---


### getTestListeners

```
public java.util.List<ITestListener> getTestListeners()
```

:   **Specified by:**: `getTestListeners` in interface `org.testng.internal.ITestResultNotifier`

---


### getConfigurationListeners

```
public java.util.List<IConfigurationListener> getConfigurationListeners()
```

:   **Specified by:**: `getConfigurationListeners` in interface `org.testng.internal.ITestResultNotifier`

---


### getVerbose

```
public static int getVerbose()
```

---


### setVerbose

```
public void setVerbose(int n)
```

---


### addListener

```
public void addListener(java.lang.Object listener)
```

---


### addTestListener

```
public void addTestListener(ITestListener il)
```

---


### getInvokedMethods

```
public java.util.List<ITestNGMethod> getInvokedMethods()
```

---


### setMethodInterceptor

```
public void setMethodInterceptor(IMethodInterceptor methodInterceptor)
```

---


### getCurrentXmlTest

```
public XmlTest getCurrentXmlTest()
```

:   **Specified by:**: `getCurrentXmlTest` in interface `ITestContext`
:   **Returns:**: the current XmlTest.

---


### getAttribute

```
public java.lang.Object getAttribute(java.lang.String name)
```

:   **Specified by:**: `getAttribute` in interface `IAttributes`
:   **Parameters:**: `name` - The name of the attribute to return

---


### setAttribute

```
public void setAttribute(java.lang.String name,
                         java.lang.Object value)
```

:   **Description copied from interface: `IAttributes`**
:   Set a custom attribute.

    :   **Specified by:**: `setAttribute` in interface `IAttributes`

---


### getAttributeNames

```
public java.util.Set<java.lang.String> getAttributeNames()
```

:   **Specified by:**: `getAttributeNames` in interface `IAttributes`
:   **Returns:**: all the attributes names.

---


### removeAttribute

```
public java.lang.Object removeAttribute(java.lang.String name)
```

:   **Description copied from interface: `IAttributes`**
:   Remove the attribute

    :   **Specified by:**: `removeAttribute` in interface `IAttributes`
    :   **Returns:**: the attribute value if found, null otherwise

---


### getGuiceModules

```
public java.util.List<com.google.inject.Module> getGuiceModules(java.lang.Class<? extends com.google.inject.Module> cls)
```

:   **Specified by:**: `getGuiceModules` in interface `ITestContext`

---


### addGuiceModule

```
public void addGuiceModule(java.lang.Class<? extends com.google.inject.Module> cls,
                           com.google.inject.Module module)
```

:   **Specified by:**: `addGuiceModule` in interface `ITestContext`

---


### getInjector

```
public com.google.inject.Injector getInjector(java.util.List<com.google.inject.Module> moduleInstances)
```

:   **Specified by:**: `getInjector` in interface `ITestContext`

---


### addInjector

```
public void addInjector(java.util.List<com.google.inject.Module> moduleInstances,
                        com.google.inject.Injector injector)
```

:   **Specified by:**: `addInjector` in interface `ITestContext`


---


|  |  |  |
| --- | --- | --- |
| **Overview** \| **Package** \| **Class** \| **Tree** \| **Deprecated** \| **Index** \| **Help** | |  |
| **PREV CLASS**   **NEXT CLASS** | **FRAMES**    **NO FRAMES**     **All Classes** |  |
| SUMMARY: NESTED \| FIELD \| CONSTR \| METHOD | DETAIL: FIELD \| CONSTR \| METHOD |  |


---
